# Using Epoxidized Solution Polymerized Styrene-Butadiene Rubbers (ESSBRs) as Coupling Agents to Modify Silica without Volatile Organic Compounds

**DOI:** 10.3390/polym12061257

**Published:** 2020-05-30

**Authors:** Chaohao Liu, Mingming Guo, Xiaobo Zhai, Xin Ye, Liqun Zhang

**Affiliations:** 1 State Key Laboratory of Organic-Inorganic Composites, Beijing University of Chemical Technology, P.O. Box 57, Beisanhuan East Road, Beijing 100029, China; 2016400091@mail.buct.edu.cn (C.L.); 2019400094@mail.buct.edu.cn (X.Z.); 2 SINOPEC Beijing Research Institute of Chemical Industry, Beijing 100013, China; guomm57@swu.edu.cn; 3 Engineering Research Center of Elastomer Materials on Energy Conservation and Resources, Ministry of Education, Beijing University of Chemical Technology, Beijing 100029, China

**Keywords:** SSBR, epoxidation, silica, VOCs

## Abstract

Rubber used in tire is usually strengthened by nanofiller, and the most popular nanofiller for tire tread rubber is nano silica, which can not only strengthen rubber but also lower the tire rolling resistance to reduce fuel consumption. However, silica particles are difficult to disperse in the rubber matrix because of the abundant silicon hydroxyl on their surface. Silane coupling agents are always used to modify silica and improve their dispersion, but a large number of volatile organic compounds (VOCs) are emitted during the manufacturing of the nanosilica/rubber composites because of the condensation reaction between silane coupling agents and silicon hydroxyl on the surface of silica. Those VOCs will do great harm to the environment and the workers’ health. In this work, epoxidized solution polymerized styrene-butadiene rubbers (ESSBR) with different epoxy degrees were prepared and used as macromolecular coupling agents aimed at fully eliminating VOCs. Fourier transform infrared (FTIR) spectroscopy and nuclear magnetic resonance (NMR) analyses verified that the different ESSBRs were successfully synthesized from solution polymerized styrene-butadiene rubbers (SSBR). With the help of the reaction between epoxy groups and silicon hydroxyl without any VOC emission, nanosilica can be well dispersed in the rubber matrix when SSBR partially replaced by ESSBR which was proved by Payne effect and TEM analysis. Dynamic and static mechanical testing demonstrated that silica/ESSBR/SSBR/BR nanocomposites have better performance and no VOC emission compared with Bis-(γ-triethoxysilylpropyl)-disulfide (TESPD) modified silica/rubber nanocomposites. ESSBR is very hopeful to replace traditional coupling agent TESPD to get high properties silica/rubber nanocomposites with no VOCs emission.

## 1. Introduction

In recent years, with the stricter requirements for tire performance, shortage of petroleum resources, and people’s attention to environmental protection, better wet-skid resistance property as well as lower rolling resistance are demanded when rubber is applied to tire tread [1,2,3]. In this context, the concept of “green tires” was proposed by Michelin in 1992, which refers to a compound composed of SSBR, silica and some other reinforcing agents [4,5].

However, there is a problem when silica is used to strengthen the rubber. Silica is a general term for fine powdery or superfine particle precipitated silica. There is a mass of activated silicon hydroxyl groups on the surface of silica [6,7,8] which makes it hard to infiltrate and disperse in the organic rubber phase and easy to aggregate by itself [9,10]. 

In the 1970s, it was found the silica ability to be modified by silane coupling agents such as bis-(γ-triethoxysilylpropyl)-tetrasulfide (TESPT) or bis-(γ-triethoxysilylpropyl)-disulfide (TESPD) which can improve the compatibility between silica and rubber [11,12,13]. In this process, the silane coupling agent reacts with the hydroxyl on the surface of silica and makes the silica turn from hydrophilic to hydrophobic [14,15]. It usually requires a high temperature (150 °C) for this reaction when the mixing of rubber compounds begins. Inevitably, volatile organic compounds (VOCs) [16,17,18,19] like methanol and ethanol will be produced during this process [20,21], which is harmful to rubber performance and worker’s health [22,23].

To improve the performance of silica/rubber nanocomposites and lower the VOCs emission, as opposed to modifying the silica surface, the compatibility of silica and rubber can also be improved by the functionalization of the rubber molecular chain, e.g., introducing functional groups like carboxyl groups [24,25], hydroxyl groups [26,27,28,29,30], alkoxysilane groups, and epoxy groups [3,20,28] onto rubber chains during post-polymerization process. Additionally, these functional groups, especially alkoxysilane groups [31,32,33,34], can be introduced to the polymer chain during the polymerization process, copolymerization, or termination. These groups provide the rubber with polarity or more reactivity with silicon hydroxyl on silica surface. However, some of alkoxysilane groups are likely to emit VOCs because the processing temperature of rubber and silica is always high (150 °C) and the alcohol products with low molecular weight will turn into gases. Therefore, the other groups are more environmentally friendly. Jacobi et al [35,36] have synthesized ESSBR and elaborated the relationship of epoxy degree with double bond content, hydrogen peroxide concentration, reaction temperature and time. However, the reaction between epoxy groups and hydroxyl groups was rarely reported [20,37], which is a ring opening reaction without any VOCs. Therefore, we plan to use the ESSBR as macromolecule coupling agents to modify the performance of silica/rubber nanocomposites and eliminate the VOCs emission.

In this study, a series of ESSBR with different epoxy degrees (7%–25%) were prepared by using formic acid and hydrogen peroxide as oxidant, and silica/ESSBR/SSBR/BR nanocomposites were manufactured. SSBR matrix were partially replaced by ESSBR with different epoxy degrees (7%–25%) as macromolecule coupling agents. The performance of silica/ESSBR/SSBR/BR composites were examined by transmission electron microscopy (TEM), rubber process analyzer (RPA), tension tester, and dynamic mechanical thermal analysis (DMTA). 

## 2. Experiment

### 2.1. Materials

Solution polymerized styrene-butadiene rubber 2557 (SSBR2557) was supplied by SINOPEC Lanzhou Petrochemical Co. Ltd. (Lanzhou, China). Solution polymerized styrene-butadiene rubber 4526 (SSBR4526) and butadiene rubber (CB24) were purchased from LANXESS Corporation (Shanghai, China). Cyclohexane was obtained from Beijing Chemworks Company (Beijing, China). Hydrogen peroxide (30%) was obtained from Shanghai Chemical Reagent Company (Shanghai, China). Formic acid was obtained from Beijing TG fine chemicals Company (Beijing, China). Tween-80 was obtained from Beijing Yili Fine Chemicals Company (Beijing, China). All of the rubber additives, including zinc oxide, stearic acid, N-Isopropyl-N’-phenyl-4-phenylenediamin (antioxidant 4010NA), wax, N-cyclohexyl-2-benzothiazole sulfonamide (accelerator CZ), and sulfur, were industrial grade and commercially available.

### 2.2. Preparation of Epoxidized Styrene-Butadiene Rubber (ESSBR)

**Figure 1:**
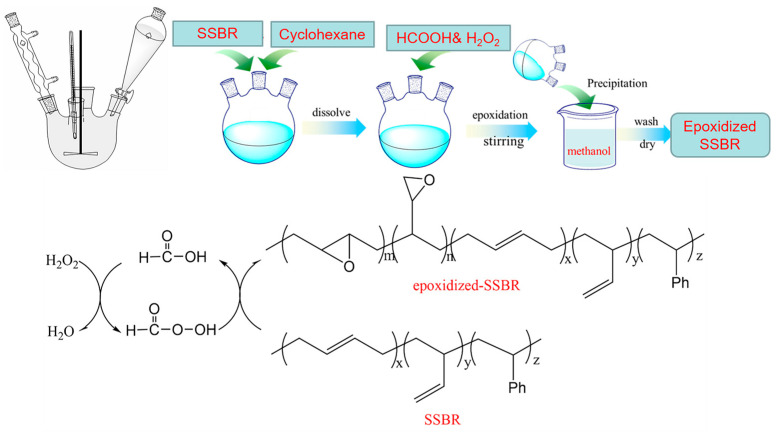
Reaction principle and flow chart.

Cyclohexane was added to a three-necked flask. The SSBR was then added at a mass-to-volume ratio of 10 g/100 mL. The mixture was stirred until the rubber was dissolved. The temperature was set at 40 °C. Hydrogen peroxide was used in excess of the polymers double bond content (H_2_O_2_/C=C 1.5/1), and formic acid was added at a ratio of the reactant mol (H_2_O_2_/HCOOH 3/1) to generate in situ performic acid. The mixing was done at 50 rpm rotor speed with a mechanical stirrer. Formic acid was all added one time, and hydrogen peroxide was added by the rate of 3 mL/min with an addition funnel. At the end of the reaction, the mixture was neutralized with a (5% w/v) Na_2_CO_3_ solution and washed with distilled water. After removing the aqueous phase, the organic phase was coagulated in ethanol and dried under vacuum to a constant weight. The reaction principle and flow of ESSBR preparation was described in Figure 1.

In order to prepare ESSBR with different epoxy degree, the amount of reactants were constant: SSBR 100 g, hydrogen peroxide (30%) 180 g, formic acid 24.4 g. And the reaction time changed: ESSBR7% (28 min), ESSBR10% (35 min), ESSBR15% (68 min), ESSBR20% (117 min), ESSBR25% (264 min).

### 2.3. Preparation of Silica/SSBR/BR Composites

**Figure 2:**
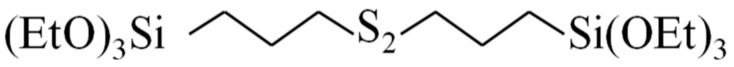
The molecular structure of bis-(γ-triethoxysilylpropyl)-disulfide (TESPD).

In our earlier research, we used pure ESSBR to replace SSBR. When the epoxy degree of pure ESSBR is more than about 7%, a crushing phenomenon of rubber compounds appears during the mixing process. When blends of SSBR and ESSBR are used, this issue can be solved. ESSBR with different epoxy degrees (7%–25%) were used as macromolecular coupling agents to modify the silica surface and improve its dispersion. Bis-(γ-triethoxysilylpropyl)-disulfide (TESPD) shown in Figure 2, which is the most popular silane coupling agent used in tire factories, was selected for comparison. All the silica/SSBR/BR compounds are listed in Table 1. Firstly, silica, antioxidants, wax, zinc oxide, stearic acid, and TESPD or ESSBR were mixed with SSBR/BR in a Haake internal mixer at 50 °C by the standard adding sequence. Secondly, the compounds were masticated for 5 min at 150 °C, then the compounds were taken out, cooled down to room temperature, and mixed with the vulcanization accelerators and sulfur on a 6-inch two-roll mill for 5 min at room temperature. Finally, the compounds were cured under 15 MPa at 150 °C to yield the vulcanized nanocomposite.

### 2.4. Characterizations

Fourier transform infrared (FT-IR, Bruker Tensor-27 FT-IR Spectrometer, Bruker Optik Gmbh Co., Ettlingen, Germany) measurement was used to identify the groups in ESSBR, using attenuated total reflection(ATR) mode under a wave ranging from 400 to 4000 cm^−1^ with 32 scans. 

The ^1^H NMR spectroscopy were recorded on a Bruker AV400 spectrometer and CDCl_3_ was the solvent, using a concentration of 7–20 mg polymer/mL.

Differential scanning calorimetry (DSC) measurements were conducted using a STARe system DSC instrument from −100 to 100 °C with a heating rate of 10 °C min^−1^ under nitrogen.

The molecular weights of rubbers were obtained by gel permeation chromatography (GPC) on an Agilent 1260 Infinity instrument equipped with a G1362A refractive index detector. Toluene was the mobile phase (1.0 mL min^−1^), and polystyrene standards were used for calibration.

The curing behavior of silica/SSBR/BR composites were measured by a MR-C3 rotorless rubber vulcanizing machine at 150 °C and 1.67 Hz.

The silica dispersion was observed under a Tecnai G220 TEM (FEI Co., Hillsboro, OR, USA) with an accelerating voltage of 200 kV. The thin sections of silica/SSBR/BR nanocomposites were cut for TEM observations using a microtome at −100 °C and collected on copper grids.

The bound rubber contents of the silica/SSBR/BR composites were measured based on the previously reported method [38].

A Bruker AVANCE III 400 WB solid-state NMR spectrometer was used to characterize the crosslink density of silica/SSBR/BR composites. The sample was packed into a 10 mm diameter NMR tube and then moved to the heating zone of the nuclear magnetic instrument with the same temperature as the oven. The sample was stabilized for 5 min and then scanned at 90 °C.

The dynamic rheological properties of the silica/SSBR/BR composites were analyzed by RPA 2000 (Alpha Technologies Co., Bellingham, WA, USA) at 60 °C and 1 Hz (mainly to get tanδ@60 °C). For compounds, the strain amplitude was varied from 0.1% to 450%. For cured composites, the strain amplitude was varied from 0.1% to 100%.

The thermo-mechanical properties of the nanocomposites were analyzed by a 01dB-Metravib VA 3000 dynamic mechanical thermal analyzer (DMTA) at 10 Hz in the tension mode with a strain amplitude of 0.1%. The test temperature ranged from −80 to 80 °C with a heating rate of 5 °C min^−1^. (mainly to get tanδ@0 °C).

The mechanical properties of the silica/SSBR/BR composites were investigated according to ASTM D638 specifications using a CMT4104 electrical tensile tester (Shenzhen SANS Test Machine Co., Shenzhen, China) at across head speed of 500 mm/min.

The abrasion loss properties of the silica/SSBR/BR composites were measured due to GB/1689–1998 with a MZ-4061 Akron abrasion machine (Jiangsu Mingzhu Experimental Machinery Co. LTD., Yangzhou, CN).

## 3. Results and Discussion 

### 3.1. Chemical Structure of the Epoxidized Styrene-Butadiene Rubber (ESSBR)

**Figure 3:**
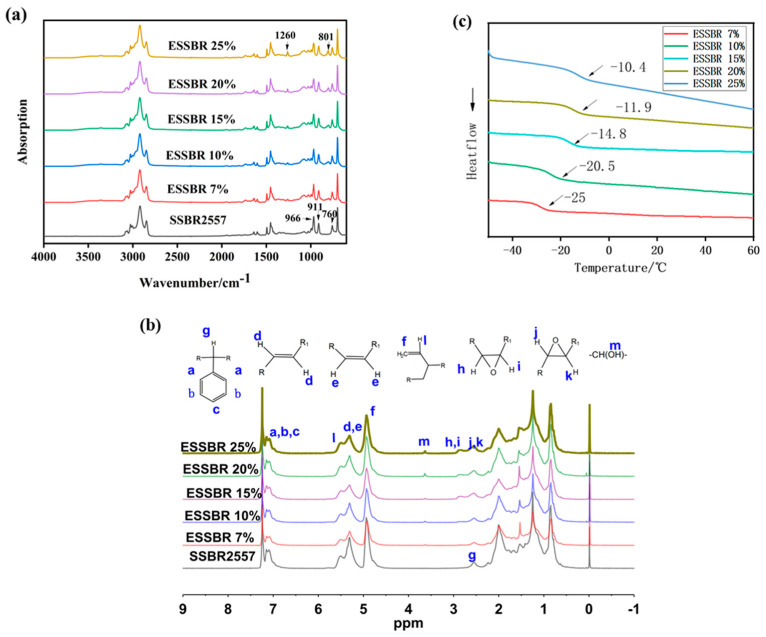
Structure characterization of SSBR and epoxidized solution polymerized styrene-butadiene rubbers ESSBRs: (**a**) FT-IR spectra (**b**) ^1^H NMR spectra and (**c**) DSC.

Figure 3a shows the FT-IR spectra of the synthesized product. Peaks at 760 cm^−1^ are related to 1,4-cis, those at 966 cm^−1^ to 1,4-trans, and those at 911 cm^−1^ to 1,2-vinyl double bonds, respectively. The new peaks at 1260 and 801 cm^−1^, which refer to the symmetrical stretching deformation absorption peak of C–O–C and the asymmetric extension deformation vibration absorption peak of C–O–C, respectively, can be observed from the ESSBR curve, indicating that the epoxy groups have been introduced into the molecular chain of the SSBR.

As shown in Figure 3b, H atoms in SSBR and ESSBR are marked as a–k according to their local chemical environment. The peaks shown reasonably correspond to the protons of styrene, butadiene (including 1,4-addition and 1,2-addition), and the epoxy group. In the ^1^H NMR spectrum of SSBR2557, there are two peaks at 4.90 (f,l) and 5.25 (d.e)ppm, attributed to the protons of the double bonds 1,2 and 1,4 butadiene units (cis and trans) in the polymer. In the ^1^H-NMR spectrum of ESSBR, three new peaks appear at 2.55 (j,k), 2.80 (h,i), and 3.64 ppm (m,). The peaks at 2.55 (j,k) and 2.80 (h,i) ppm correspond to the methine resonance of the epoxy groups in trans and cis position, respectively, which demonstrate that SSBR was successfully epoxidized. The peaks at 3.64 (m,) derive from hydroxyl groups [29] which formed during ring-opening reactions after epoxidation and the wide bands in 3100–3600 cm^−1^ from FT-IR also indicates the presence of hydroxyl groups, but the amount of hydroxyl groups was few and the epoxy degree was little affected. As the reaction progresses, the epoxy degree increases, resulting in an increase of the signals at 2.55 (j,k) and 2.80 (h,i) ppm (trans and cis epoxy) and a decrease of the peak at 5.25 (d.e) ppm (unsaturated 1,4-polybutadiene protons). The peak at 4.9 (f,l) ppm (vinyl group) remains practically constant. These findings indicated that the reactivity of trans and cis 1,4 units is higher than that of vinyl 1,2 units. the epoxy degree, *X*, for SSBR has been calculated using the following Equation:

$$X\%=\left(\frac{A_{epox}-A_{H_{est}}}{\left(A_{epox}-A_{H_{est}}\right)+A_{1,4}+A_{1,2}}\right)\times 100$$

where *A_epox_* is the normalized proton area intensities for the epoxide peaks at 2.55 and 2.80 ppm, *A_H_est__* is the normalized area intensities for methylene bonded to the styrene ring at 2.55 ppm, *A*_1,4_ and *A*_1,2_ are the normalized area for the unsaturated 1,4 polybutadiene peak at 5.25 ppm and unsaturated 1,2 polybutadiene peak at 4.9 ppm.

As shown in Figure 3c, when the epoxy degree increases, the polarity of macromolecules increases, and the *T*_g_ (glass transition temperature) increases which results in tanδ@0 °C. This is helpful for improving the wet-skid resistance of rubber composites below. 

ESSBR with the epoxy degree from 7% to 25% were prepared and got ready to use as macromolecule coupling agents, because the epoxy groups can react with the silicon hydroxyl on silica surface, and ESSBR has good compatibility with the SSBR/BR matrix, the residual double bonds on ESSBR can also crosslink with SSBR/BR matrix by vulcanizing agent.

### 3.2. Compositions and Molecular Weights of Rubbers

From Table 2, it can be seen that the ESSBRs have a little increase about *M*_n_ and *M*_w_ and similar PDI (Polymer dispersity index) with SSBR2557 after epoxidation, which means they have consistent molecular weight distribution, the side reaction of epoxidation is less and the reaction is controllable. 

### 3.3. Application of ESSBR in Rubber Composites

#### 3.3.1. Payne Effect of Silica/SSBR/BR Compounds

**Figure 4:**
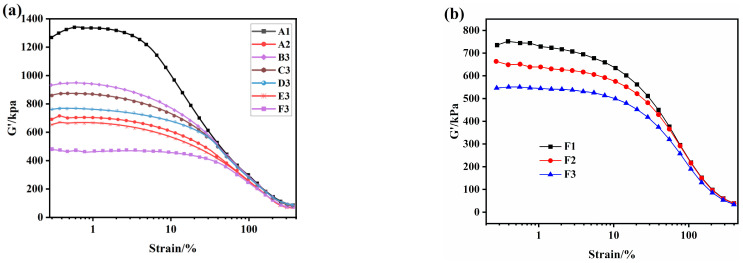
Strain amplitude dependence of the storage modulus (G’) of silica/SSBR/BR compounds: (**a**) samples with different ESSBRs(epoxy degree), (**b**) samples with different content of E25.

The strain amplitude dependence of the storage modulus (G’) of silica/SSBR/BR compounds are shown in Figure 4. The filler’s network and its situation of agglomeration make a great influence on the modulus of rubber compounds. The G’ decreases rapidly with the increase of strain amplitude, named the Payne effect [39,40], which is closely related to the breakdown of the filler network structure in rubber matrix when the deformation rate of specimen increases. The difference between the maximum and the minimum G’ in the curve names the ΔG’ value, which is usually negatively correlated to the dispersion of filler. The lower the ΔG’ value, the better the filler disperses. 

As shown in Figure 4a, PS-SSBR/BR(A1) has a significant Payne effect because there is not any modifier for polar silica, therefore, silica particles couldn’t be well dispersed in the nonpolar rubber matrix and so the silica particles agglomerated by themselves. With part of the SSBR replaced by ESSBR, the epoxy groups on the macromolecular chains could react with silicon hydroxyl groups on the silica surface and reduce the polarity of silica particles, which could greatly improve the silica dispersion in rubber matrix [41,42]. That means that the silica–silica direct contact networks reduced and more silica–rubber networks were established and then the ΔG’ decreased. It is clear that the Payne effects of ESSBR-silica samples and TESPD-silica sample are significantly less than that of the pure silica sample. With the epoxy degree increase, the Payne effect of the composites decrease (B3 to F3). When the epoxy degree is fixed, ΔG’ decreased as the amount of ESSBR increased (F1 to F3). All the results show that the introduction of epoxy groups on SSBR is very beneficial to improve the silica dispersion. ΔG’ of E20-SSBR/BR (E3) and E25-SSBR/BR (F3) is even less than that of TS-SSBR/BR(A2). This result indicates that ESSBR used as a modifier has similar or even better effects on improving the dispersion of silica in silica/SSBR/BR compounds when comparing with TESPD, because the reaction between epoxy groups on ESSBR and silicon hydroxyl groups on the silica surface can reduce the hydrophilicity of silica and improve the compatibility between silica and rubber.

#### 3.3.2. TEM Images of Silica/SSBR/BR Composites

**Figure 5:**
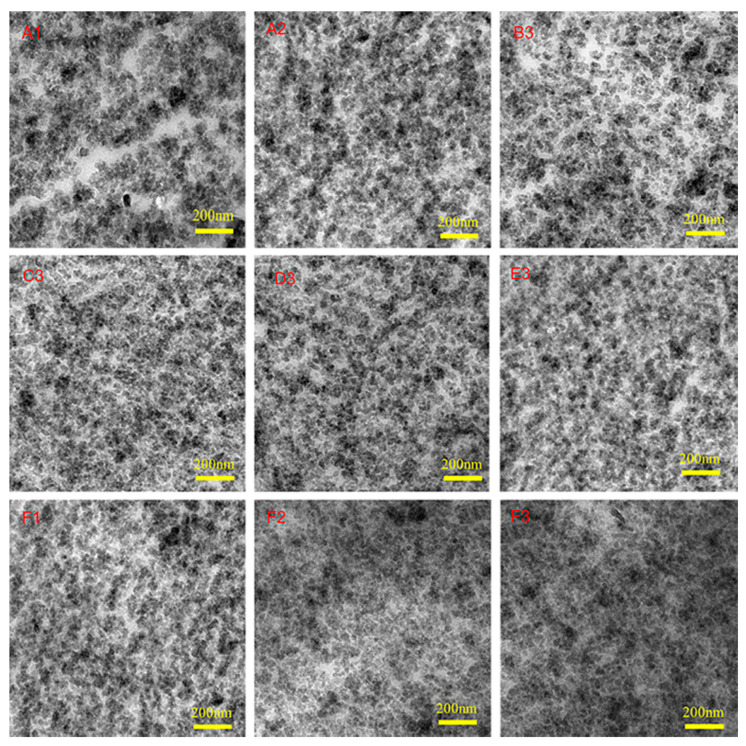
TEM images of PS-SSBR/BR(**A1**), TS-SSBR/BR(**A2**), E7-SSBR/BR(**B3**), E10-SSBR/BR(**C3**), E15-SSBR/BR(**D3**), E20-SSBR/BR(**E3**), E25a-SSBR/BR(**F1**), E25b-SSBR/BR(**F2**) and E25c-SSBR/BR(**F3**).

Figure 5 are the TEM images which show the dispersion of silica in rubber matrix. The darker phase represents the silica particles. Silica particles are obviously agglomerated in the PS-SSBR/BR composite and forms a lot of clumps. Moreover, the phase interface between silica particles and the rubber matrix looks obviously clear. As is known to all, there is a layer of silicon hydroxyl distributed evenly on the surface of silica particles leading to silica agglomerate. With part of the SSBR replaced by ESSBR, the dispersion of silica particles was obviously improved. 

It can be seen from Figure 5 that the silica in E25-SSBR/BR (F3) has the best dispersion, even dispersing better than silica in TS-SSBR/BR(A2). The dispersion of silica in E15-SSBR/BR (D3) and E20-SSBR/BR (E3) is comparable to that of TS-SSBR/BR(A2). Previous studies [43,44,45] have indicated that the chemical interaction between silica and rubber can make its dispersion in the rubber matrix more stable, as silica is not easy to aggregate again because of the stability of the chemical interaction between silica and rubber. TESPD has the ability to create the chemical interaction between the silica surface and rubber. On the one hand, ESSBR can make chemical interactions with silica, on the other hand, it can be well mixed with rubber matrix. Similar to TESPD, ESSBR can also improve the dispersion of silica in the rubber matrix by forming a chemical interaction between silica and rubber.

As shown in Figure 5, when the ratio of SSBR replaced by ESSBR is 40% (B3,C3,D3,D3,F3), the dispersion of silica improves with the increase of the epoxy degree of ESSBR. Additionally, when the epoxy degree of ESSBR used remains at 25% (F1,F2,F3), the dispersion of silica improved with the increase of the ratio of ESSBR/SSBR.

#### 3.3.3. Bound Rubber of Silica/SSBR/BR Composites

**Figure 6:**
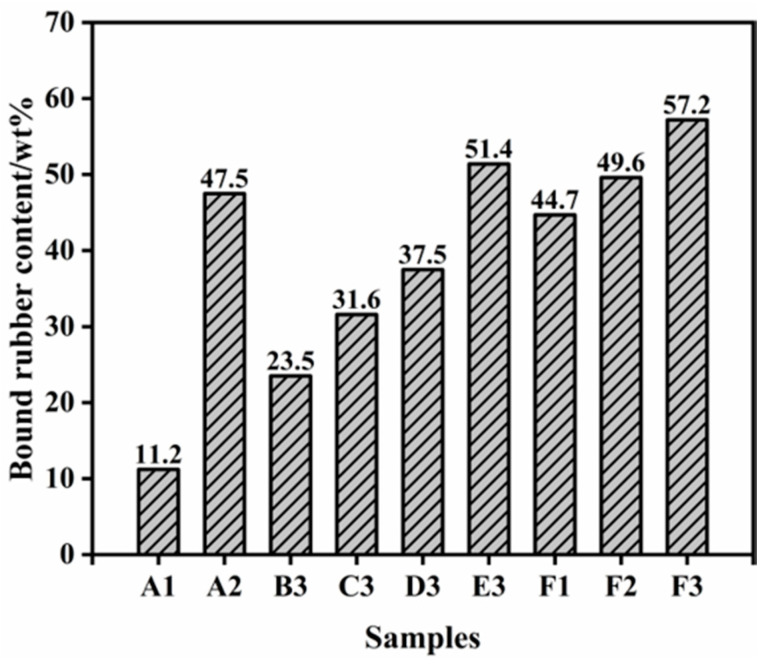
Bound rubber content of PS-SSBR/BR, ESSBR-SSBR/BR and TS-SSBR/BR composites.

Bound rubber, which is the adsorbed rubber on the filler surface, is influenced by the interfacial interaction between the filler and rubber. The bound rubber contents of the silica/SSBR/BR compounds were measured, as shown in Figure 6. When the ratio of SSBR replaced by ESSBR is 40%, it can be seen that the bound rubber content increases with the increase of the epoxy degree of ESSBR. As more epoxy groups react with silica [41,42], the hydrophilicity of silica decreases and the interaction between silica and rubber improves. It also can be seen that when the epoxy degree of ESSBR used remains at 25%, with the increase of the ratio of ESSBR/SSBR, the bound rubber content increases.

#### 3.3.4. Dynamic Mechanical Properties of Silica/SSBR/BR Composites

When rubber nanocomposite is applied to tire treads, the anti-wet skid performance is generally correlated with the tanδ values at 0 °C [46,47]. The higher the tanδ value at 0 °C is, the better the anti-skid performance is. Additionally, the rolling resistance of tires is correlated with the tanδ values at 60 °C [48,49], the lower the tanδ value at 60 °C is, the lower the rolling resistance is. 

**Figure 7:**
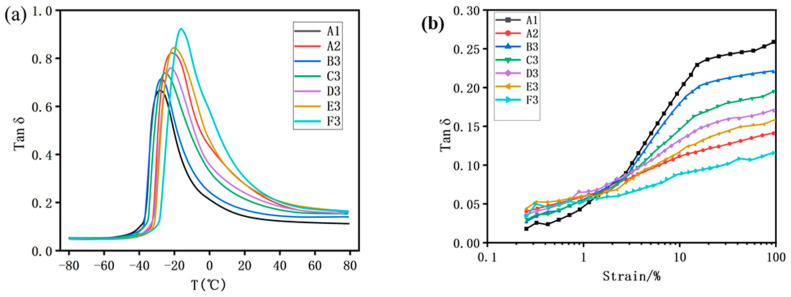
Dynamic properties of PS-SSBR/BR, ESSBR-SSBR/BR and TS-SSBR/BR composites: (**a**) the relation of the tanδ with temperature and (**b**) the relation of the tanδ with strain amplitude.

From Figure 7a, when the ratio of SSBR replaced by ESSBR is 40%, it can be seen that with the increase of the epoxy degree of ESSBR, the glass transition temperature of rubber gradually increases [50,51]. As the rigidity of the molecular chain enhances after the increase of epoxy groups on the rubber molecule, the internal rotation hindrance of the molecular chain increases and the activity decreases. It can be seen from Table 3 that with the increase of the epoxy degree of ESSBR, the tanδ value at 0 °C increases, indicating that the anti-wet skid performance increases. The tanδ values at 0 °C of E20-SSBR/BR (E3) and E25-SSBR/BR (F3) exceeds that of TS-SSBR/BR. This result indicates that ESSBR is very useful to improve the wet-skid resistance performance of the tire tread and its effect is better than TESPD when the epoxy degree is up to 20%.

As shown in Figure 7b, the tanδ values at 60 °C of ESSBR-SSBR/BR composites and TS-SSBR/BR are both lower than that of PS-SSBR/BR. The rolling resistance of PS-SSBR/BR is significantly higher than that of ESSBR-SSBR/BR and TS-SSBR/BR. The above situation happens mainly because there exists strong mutual friction between silica particles under cyclic reversed loading in PS-SSBR/BR. In contrast, for ESSBR-SSBR/BR and TS-SSBR/BR composites, this friction loss between silica particles decreased due to the chemical interaction between silica and rubber molecular and less silica-silica interaction. It can be seen from Table 3 that with the increase of epoxy degree of ESSBR, tanδ values at 60 °C decreases, indicating that the rolling resistance of tires decreases. The tanδ values at 60 °C of E25-SSBR/BR (F3) is lower than that of TS-SSBR/BR and tanδ values at 60 °C of E20-SSBR/BR (E3) is similar to that of TS-SSBR/BR. This result indicates that ESSBR is beneficial for lowering the rolling resistance of tire tread and its effect is better than TESPD when the epoxy degree is up to 20%.

**Figure 8:**
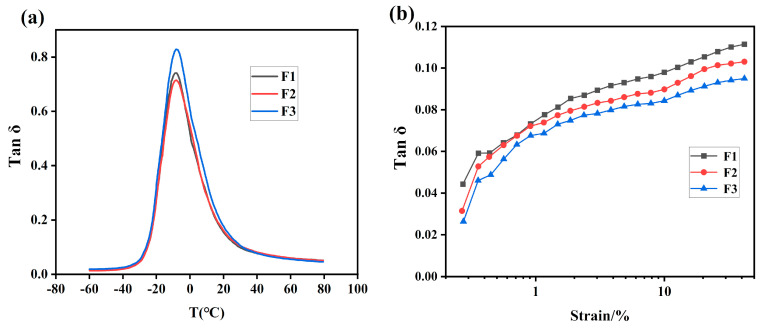
Dynamic properties of E25-SSBR/BR composites with different ratio of ESSBR/SSBR: (**a**) the relation of the tanδ with temperature and (**b**) the relation of the tanδ with strain amplitude.

Table 4 and Figure 8a are the dynamic properties of E25-SSBR/BR composites with different ratios of ESSBR/SSBR. It can be seen that when the epoxy degree of ESSBR used remains up to 25%, with the increase of ratio of ESSBR/SSBR, the glass transition temperature of rubber increases. The tanδ values at 60 °C of E25-SSBR/BR composites decreases with the increase of ratio of ESSBR/SSBR, indicating that the rolling resistance of tires decreases.

#### 3.3.5. Curing Behavior of Silica/SSBR/BR Composites

When the ratio of SSBR replaced by ESSBR is 40%, it can be seen from Table 5 that with the increase of the epoxy degree of ESSBR, *T*_10_ (the time when the torque reaches 10% of the maximum torque) increases. It was speculated that the epoxy group reacted with the promoter during the mixing process, which weakened the effect of the promoter, *T*_90_ (the time when the torque reaches 90% of the maximum torque) tends to decrease slightly. This is possibly because during the vulcanization process the epoxy group increases the reactivity of adjacent double bonds, thus the crosslinking time had been shortened. *M*_L_ (the minimum torque) is related to filler-filler network, with the increase of the epoxy degree of ESSBR, filler-filler network is weakened and *M*_L_ decreases. *M*_H_ (the maximum torque) is influenced by filler–filler network, filler–rubber network, and rubber–rubber network, and there is no obvious rule to its changing. An optimal crosslink density is very important to achieve rubber with good mechanical properties, with the increase of the epoxy degree of ESSBR, crosslink density tends to increase.

#### 3.3.6. Static Mechanical Properties of Silica/SSBR/BR Composites

**Figure 9:**
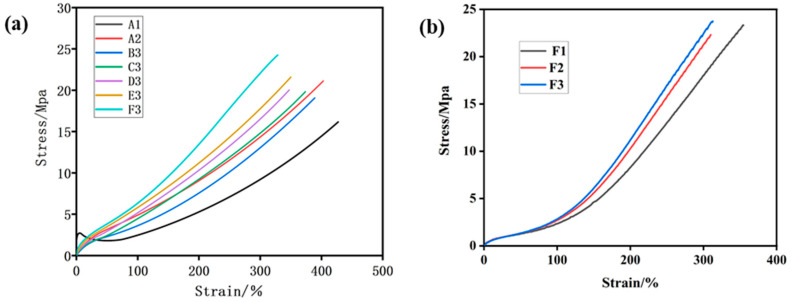
Mechanical properties of silica/SSBR/BR composites: (**a**) samples with different ESSBRs(epoxy degree), (**b**) samples with different content of E25.

When the ratio of SSBR replaced by ESSBR is 40%, it can be seen from Figure 9 and Table 6 that with the increase of the epoxy degree of ESSBR, the modulus at 100% and 300% strain, the tensile stress of vulcanized rubber increases, and the elongation at break decreases. The tensile strength is related to the crosslinking density and the interaction between filler and rubber matrix. The higher the epoxy degree is, the better the binding force of silica with the molecular chain is. This binding force limits the movement of the molecular chain, thereby the tensile strength and the modulus of constant elongation increases, and the elongation at break decreases.

It also can be seen from Figure 9 and Table 6 that when the epoxy degree of ESSBR used remains up to 25%, with the increase of ratio of ESSBR/SSBR, the Modulus at 100% and 300% strain of vulcanized rubber increases, the elongation at break decreases. 

#### 3.3.7. Abrasion Loss Properties of Silica/SSBR/BR Composites

**Figure 10:**
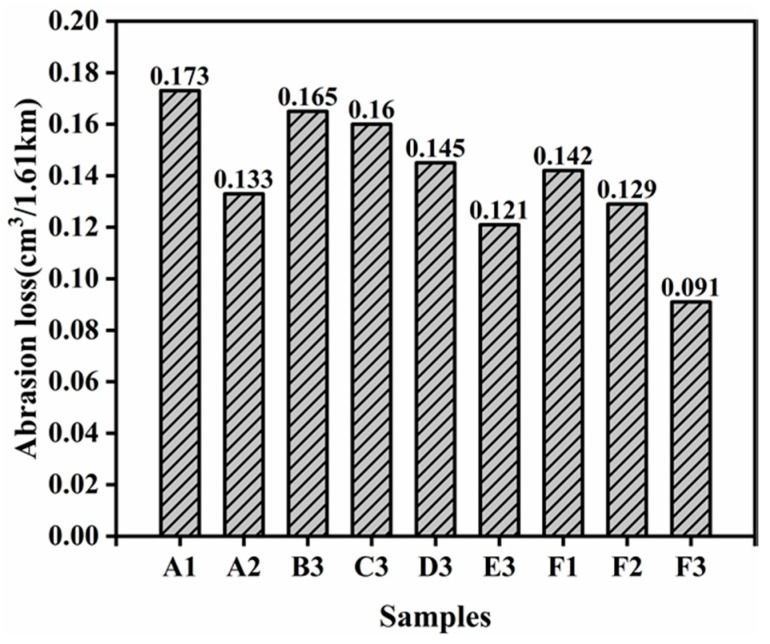
Abrasion loss properties of silica/SSBR/BR composites.

When the ratio of SSBR replaced by ESSBR is 40%, it can be seen from Figure 10, with the increase of the epoxy degree of ESSBR, the abrasion volume decreases gradually, due to more epoxy groups reacting with silica and creating more rubber–filler chemical interaction, which may be helpful to improve wear-resisting properties. It also can be seen that when the epoxy degree of ESSBR used remains up to 25%, with the increase of ratio of ESSBR/SSBR, the abrasion volume decreases.

## 4. Conclusions

In this research, SSBR was epoxidized to ESSBR with different epoxy degrees and then used as a macromolecular coupling agent to modify silica/rubber nanocomposites. Due to the ring- opening reaction between epoxy groups and the silicon hydroxyl without any VOCs emission, silica/rubber nanocomposite for tire tread can be made with no VOC emission. Additionally, as ESSBR has good compatibility with SSBR/BR matrix and can be crosslinked with the rubber matrix, silica–ESSBR–rubber matrix chemical bonds can be formed. As a result, ESSBR as a macromolecular coupling agent is beneficial for silica/SSBR/BR nanocomposites used for green tire treads to get better wet-skid resistance and lower rolling resistance, with no VOC emission. It is a hopeful candidate to replace the traditional coupling agent, TESPD, which has VOCs emission.

## Figures and Tables

**Table 1:** Formulation of silica/ solution polymerized styrene-butadiene rubber (SSBR)/ butadiene rubber (BR) compounds.

	A1	A2	B3	C3	D3	E3	F1	F2	F3
Materials	PS(pure silica)-SSBR/BR/phr ^a^	TS(TESPD-silica)-SSBR/BR/phr ^a^	E7-SSBR/BR/phr ^a^	E10-SSBR/BR/phr ^a^	E15-SSBR/BR/phr ^a^	E20-SSBR/BR/phr ^a^	E25a-SSBR/BR/phr ^a^	E25b-SSBR/BR/phr ^a^	E25c-SSBR/BR/phr ^a^
SSBR	74	74	44.4	44.4	44.4	44.4	59.2	51.8	44.4
BR	26	26	26	26	26	26	26	26	26
Silica	60	60	60	60	60	60	60	60	60
ESSBR7% ^c^	0	0	29.6^c^	0	0	0	0	0	0
ESSBR10% ^c^	0	0	0	29.6^c^	0	0	0	0	0
ESSBR15% ^c^	0	0	0	0	29.6^c^	0	0	0	0
ESSBR20% ^c^	0	0	0	0	0	29.6^c^	0	0	0
ESSBR25% ^c^	0	0	0	0	0	0	14.8	22.2	29.6^c^
TESPD	0	6	0	0	0	0	0	0	0
Other additives	1# ^b^	1# ^b^	1# ^b^	1# ^b^	1# ^b^	1# ^b^	1# ^b^	1# ^b^	1# ^b^

^a^ Parts per hundred of rubber. ^b^ 1# stearic acid 2.0, zinc oxide 3.0, N-Isopropyl-N’-phenyl-1,4-phenylenediamine 2.0, N-Cyclohexyl-2-beozothiazole sulfenamide 1.5, 1,3-Diphenylguanidine 2.0 and sulfur 1.5. ^c^ 7%–25% indicates epoxy degree of ESSBR. ^c^ the ratio of SSBR replaced by ESSBR is 40%.

**Table 2:** Compositions and molecular weights of SSBR2557, SSBR4526, CB24, and ESSBRs.

Sample	Composition (%)			*M*_n_ × 10^−5^ (Da)	*M*_w_ × 10^−5^ (Da)	PDI
	Bound Styrene	1,4-unit	1,2-unit			
SSBR2557	27	44	56	3.96	9.06	2.28
SSBR4526	26	55	45	1.75	5.4	3.09
CB24	-	-		1.45	4.05	2.79
ESSBR7%	-	-		4.35	10.11	2.32
ESSBR10%	-	-		4.48	10.53	2.35
ESSBR15%	-	-		4.58	10.68	2.33
ESSBR20%	-	-		4.31	10.42	2.42
ESSBR25%	-	-		4.46	10.71	2.40

**Table 3:** tanδ at 0 °C, 60 °C and *T*_g_ (glass transition temperature) of PS-SSBR/BR, ESSBR-SSBR/BR and TS-SSBR/BR composites.

	A1	A2	B3	C3	D3	E3	F3
tanδ@0 °C	0.213	0.433	0.246	0.324	0.366	0.467	0.605
tanδ@60 °C	0.167	0.105	0.127	0.119	0.108	0.103	0.079

**Table 4:** tanδ at 0 °C and 60 °C of E25-SSBR/BR composites with different ratio of ESSBR/SSBR.

	F1	F2	F3
tanδ@0 °C	0.537	0.568	0.627
tanδ@60 °C	0.096	0.088	0.083

**Table 5:** Curing behavior of silica/SSBR/BR composites.

	*T*_10_ (min)	*T*_90_ (min)	*M*_L_ (dNm)	*M*_H_ (dNm)	Δ*M* (dNm)	Crosslink Density (10^−4^ mol/cm^3^)
A1	1.5	53.4	38.9	56.3	17.4	1.12
A2	3.1	29.0	28.4	65.4	37	1.54
B3	3.7	53.0	34.6	66.3	31.7	1.53
C3	4.4	53.5	33.1	68.1	35	1.55
D3	4.8	51.2	31.7	65.6	33.9	1.58
E3	4.5	46.7	30.9	74.8	43.9	1.62
F1	6.6	47.3	31.2	76.1	44.9	1.59
F2	6.7	43.4	28.6	72.3	43.7	1.58
F3	6.2	44.6	26.7	69.7	43	1.61

**Table 6:** Mechanical properties of silica/SSBR/BR composites.

Sample	Elongationat Break (%)	Modulus at 100% (MPa)	Modulus at 300% (MPa)	Tensile Stress (MPa)	Shore A Hardness
A1	437 ± 39	2.6 ± 0.1	8.2 ± 0.3	16.5 ± 1.4	64
A2	386 ± 3	4.8 ± 0.1	13.6 ± 0.3	21.2 ± 0.3	63
B3	377 ± 7	3.9 ± 0.2	13.1 ± 0.4	18.2 ± 2.4	63
C3	355 ± 11	4.7 ± 0.1	13.8 ± 0.1	19.7 ± 2.5	62
D3	336 ± 27	4.9 ± 0.2	16.5 ± 0.4	20.3 ± 0.8	61
E3	338 ± 34	5.8 ± 0.4	17.4 ± 0.7	21.6 ± 2.5	64
F1	346 ± 29	5.5 ± 0.4	16.5 ± 0.4	21.9 ± 2.6	61
F2	309 ± 16	6.1 ± 0.5	20.3 ± 0.5	20.6 ± 1.5	64
F3	317 ± 26	6.6 ± 0.1	22.6 ± 0.3	23.9 ± 1.2	65
